# Perisylvian Arteriovenous Malformation Mimicking Carotid Cavernous Fistula: Operative Video

**DOI:** 10.7759/cureus.12297

**Published:** 2020-12-26

**Authors:** Michael Young, Ryan Johnson, Hamad Farhat

**Affiliations:** 1 Neurological Surgery, Advocate Health Care, Oak Lawn, USA; 2 Neurosurgery, Carle BroMenn Medical Center, Normal, USA; 3 Neurological Surgery, Advocate Christ Medical Center, Oak Lawn, USA

**Keywords:** brain arteriovenous malformation, carotid cavernous fistula, endovascular embolization, perisylvian, onyx embolization, venous congestion, spetzler martin

## Abstract

Brain arteriovenous malformations (AVM) commonly present for medical attention after a patient experiences a rupture that results in a focal neurologic deficit, an epileptic event, or is found incidentally on cranial imaging performed for an unrelated reason. In contrast, carotid-cavernous fistulas (CCF) can develop high-flow arteriovenous shunting with symptoms attributable to venous hypertension. We discuss a unique case of a 54-year-old female presenting with signs and symptoms suggestive of a CCF but was found to have a perisylvian AVM with an enlarged draining vein draining into the cavernous sinus. Our case report demonstrates a combined endovascular and open surgical approach to a unique presentation of a brain AVM with the resolution of ocular symptoms.

## Introduction

Various cerebrovascular pathologies can present with ocular symptoms. The most common cause of chemosis and conjunctival injection from a cerebrovascular pathology is due to a carotid-cavernous fistula [1]. However, rarely other etiologies such as arteriovenous malformations (AVM) can present with such symptoms due to venous congestion within the orbit [1]. The acuity of symptoms prompts a more expedited treatment because the increased venous congestion can lead to monocular blindness [2]. Treatment modalities for arteriovenous malformations include open surgery, endovascular treatment, and radiosurgery [3]. Low-grade Spetzler-Martin AVMs, as the case we present here, have been shown to be best treated with open surgery with preoperative partial embolization as needed [3].

## Case presentation

We present a case of a 54-year-old female who presented with right eye orbital pain, chemosis, and conjunctival injection. She was initially treated with antibiotics for suspected orbital cellulitis. She presented to the emergency department for continued symptoms despite antibiotic treatment. CT of orbits with contrast demonstrated evidence of a perisylvian arteriovenous malformation with arterial feeders from the cortical branches of the right middle cerebral artery and venous drainage out a dilated vein into the cavernous sinus. Diagnostic cerebral angiogram confirmed a right frontal perisylvian Spetzler-Martin grade 1 AVM (arteriovenous malformation) with arterial feeders from the right middle cerebral artery and venous drainage out a dilated vein draining into the cavernous sinus. Treatment was recommended for this AVM due to the young age of the patient giving her a higher lifetime risk of hemorrhage, low Spetzler-Martin grade indicating lower surgical risk, and the likelihood of the patient’s ocular symptoms being caused by the arterialized dilated vein draining into the cavernous sinus. Preoperative arterial pedicle feeder embolization was performed to assist with surgical resection. After embolization, the patient underwent a right pterional craniotomy for resection of this Spetzler-Martin grade 1 AVM. Complete resection of the AVM was performed and confirmed on a postoperative angiogram. Postoperative, the patient’s ocular symptoms improved immediately, and she was discharged home on a postoperative day 4 without neurologic deficits (Video 1).

**Video 1:** Perisylvian arteriovenous malformation mimicking carotid-cavernous fistula: operative video

## Discussion

Arteriovenous malformations (AVM) mimicking the clinical picture of a carotid-cavernous fistula (CCF) is a rarely reported phenomenon. A review of the literature documents few reported cases, none of which document a large draining vein draining directly into the cavernous sinus [1, 2, 4-6]. On examination, our patient complained of right eye pain with physical exam findings significant for chemosis and conjunctival injection. The hypothesis explaining the pathophysiology of this clinical presentation is a high-flow arteriovenous (AV) shunt into the cavernous sinus from the arterialized draining vein that resulted in increased venous pressure within the cavernous sinus, thereby decreasing the rate of venous drainage from the orbit due to a decreased pressure gradient. This decreased pressure gradient can be postulated to create an environment for venous stasis within the orbit and perpetuate the development of chemosis and conjunctival injection. In contrast with the more common clinical presentation of a CCF, our patient did not demonstrate any evidence of pulsatile proptosis, orbital bruits, or ophthalmoplegia [7].

Treatment for this AVM was justified based on her young age, placing her at a greater lifetime risk of hemorrhage, low Spetzler-Martin grade favoring surgical resection with acceptable morbidity risk, and the presence of ocular symptoms secondary to cavernous sinus pathology, placing her at an unacceptable risk of vision loss without intervention. As is becoming commonplace in AVM management, multimodality management with liquid embolic embolization is helpful in identifying feeding arteries during surgical resection, decreasing blood loss intraoperatively and, in our patient specifically, decreasing the degree of AV shunting. Our patient demonstrated improvement of her chemosis and conjunctival injection after her feeding artery embolization and further improvement after surgical resection of this AVM. She had an uneventful hospitalization and was discharged home without any neurologic deficits.

## Conclusions

While rare in the literature, our case clearly demonstrates that an AVM can develop venous drainage into the cavernous sinus and result in pathology that is similar to a CCF and other ocular pathology. As such, considering this pathologic entity should be on the differential diagnosis for a patient presenting with orbital pain, chemosis, and conjunctival injection.
